# Genome Wide Assessment of Genetic Variation and Population Distinctiveness of the Pig Family in South Africa

**DOI:** 10.3389/fgene.2020.00344

**Published:** 2020-05-07

**Authors:** Nompilo Lucia Hlongwane, Khanyisile Hadebe, Pranisha Soma, Edgar Farai Dzomba, Farai Catherine Muchadeyi

**Affiliations:** ^1^Biotechnology Platform, Agricultural Research Council, Onderstepoort, South Africa; ^2^Discipline of Genetics, School of Life Sciences, University of KwaZulu-Natal, Pietermartizburg, South Africa; ^3^Animal Production Institute, Agricultural Research Council, Irene, South Africa

**Keywords:** pigs, diversity, population structure, genetic characterization, SNP60K

## Abstract

Genetic diversity is of great importance and a prerequisite for genetic improvement and conservation programs in pigs and other livestock populations. The present study provides a genome wide analysis of the genetic variability and population structure of pig populations from different production systems in South Africa relative to global populations. A total of 234 pigs sampled in South Africa and consisting of village (*n* = 91), commercial (*n* = 60), indigenous (*n* = 40), Asian (*n* = 5) and wild (*n* = 38) populations were genotyped using Porcine SNP60K BeadChip. In addition, 389 genotypes representing village and commercial pigs from America, Europe, and Asia were accessed from a previous study and used to compare population clustering and relationships of South African pigs with global populations. Moderate heterozygosity levels, ranging from 0.204 for Warthogs to 0.371 for village pigs sampled from Capricorn municipality in Eastern Cape province of South Africa were observed. Principal Component Analysis of the South African pigs resulted in four distinct clusters of (i) Duroc; (ii) Vietnamese; (iii) Bush pig and Warthog and (iv) a cluster with the rest of the commercial (SA Large White and Landrace), village, Wild Boar and indigenous breeds of Koelbroek and Windsnyer. The clustering demonstrated alignment with genetic similarities, geographic location and production systems. The PCA with the global populations also resulted in four clusters that where populated with (i) all the village populations, wild boars, SA indigenous and the large white and landraces; (ii) Durocs (iii) Chinese and Vietnamese pigs and (iv) Warthog and Bush pig. *K* = 10 (The number of population units) was the most probable ADMIXTURE based clustering, which grouped animals according to their populations with the exception of the village pigs that showed presence of admixture. AMOVA reported 19.92%–98.62% of the genetic variation to be within populations. Sub structuring was observed between South African commercial populations as well as between Indigenous and commercial breeds. Population pairwise *F*_*ST*_ analysis showed genetic differentiation (*P* ≤ 0.05) between the village, commercial and wild populations. A per marker per population pairwise *F*_*ST*_ analysis revealed SNPs associated with QTLs for traits such as meat quality, cytoskeletal and muscle development, glucose metabolism processes and growth factors between both domestic populations as well as between wild and domestic breeds. Overall, the study provided a baseline understanding of porcine diversity and an important foundation for porcine genomics of South African populations.

## Introduction

Pigs were domesticated over 5,000 years ago, leading to the gradual and cumulative development of modern pig breeds with very distinctive phenotypes and production abilities (Zeder et al., 2006; Rothschild and Ruvinsky, 2010). Domesticated pig (*Sus Scrofa domesticus*) originated from the *Sus scrofa*, which is commonly known as the wild boar belonging to the Suidae family (Jones, 1998). This family includes species of wild pigs such as *Phacochoerus africanus* (Common warthog), *Potamochoerus larvatus* (Bush pig) and *Hylochoerus meinertzhageni* (Giant Forest hog) some that are indigenous to Africa (Jones, 1998). The Wild Boars are widely distributed covering areas such as Europe, Asia, and North Africa and were introduced as game species in all other continents including Africa (Jones, 1998; Scandura et al., 2011).

Pig breeds worldwide are either of well-defined ancestry or in certain instances crossbreds from populations of diverse origins (Amills et al., 2010). South African pig production consists of a commercial intensive sector with defined breeds and an extensive sector that is mainly associated with small-scale farmers in the rural areas. Village production system is characterized by non-descript populations raised under extensive low-input management. Commercial breeds such as the Large White, Landrace and Duroc have worldwide distribution in modern commercial farming systems including South Africa and are widely used (Amills et al., 2010). Indigenous breeds classified under *Sus indica* such as Kolbroek and Windsnyer are geographically restricted to Southern Africa (Nicholas, 1999). The Kolbroek, which is of Chinese origin, is speculated to have pigs that ended up in the hands of South African farmers when a sailing ship wrecked at the Cape Hangklip (Ramsay et al., 1994). Although the origin of the Windsnyer is unknown, there are observed similarities to Chinese breeds (Nicholas, 1999) thereby suggesting that it is of Chinese origin. Regardless of their origins and domestication routes, pig breeds in South Africa have become closed genetic pools restricted to specific farming systems and molded by artificial selection and possibly genetic drift (Amills et al., 2010). In addition to these domesticated breeds are the Warthog, Bush pig and Red River Hog wild pigs that are native to Africa and are found roaming in forests or in the zoos (Porter, 1993). The common Warthog (*Phacochoerus Africanus*) which was first discovered at Cape Verde, Senegal is one of the three species found in Africa. The Cape Warthog (*Phacochoerus aethiopicus*) is now extinct due to the rinderpest epizootic of the 1860s (Pallas, 1766; Gmelin, 1788; D’Huart and Grubb, 2003). Another Warthog (*Phacochoerus delamerei*) species was described in Somalia and later renamed *Phacochoerus aethiopicus delamerei* as it is similar to the Cape Warthog (Lönnberg, 1908, 1912; Roosenvelt and Heller, 1915). Muwanika et al. (2003) studied the phylogeography of the common Warthog in Africa and found three clades representing West, South and East African Warthogs. There is no enough evidence to support the origin of the Bush pig, which was assumed to have originated from Asia (White and Harris, 1977). There are recordings of the Bush pig in the Swellendam and Outeniqualand in the Western Cape provinces of South Africa (Rookmaaker, 1989). Hybrids between the domestic and Bush pigs have been recorded with the introduction of Bush pigs to South Africa being as far as 1400 years ago (Linnaeus, 1758; Mujibi et al., 2018). The existence of hybrids is a concern, as they could become asymptomatic carriers of diseases such African swine fever (Jori and Bastos, 2009).

Indigenous breeds are often geographically restricted and harbor unique genetic variants that may provide future breeds with the flexibility to change in response to product market preferences and production environments. While low-input and indigenous breeds may not compete with exotic breeds in terms of production performance, they are considered hosts to unique genetic diversity that should be protected as sources of variation. Local pigs are important because of their hardiness and ability to survive in extreme conditions (Taverner and Dunkin, 1996; Zadik, 2005). Most indigenous breeds are, however, threatened by small and fragmented flock sizes, which predispose them to lose genetic diversity as a result of genetic drift and indiscriminate crossbreeding with exotic germplasm that can lead to genetic erosion and the eradication of the local genetic pool. Globally, 35% of pig breeds are classified as at risk or already extinct (FAO, 2009) demonstrating the threat to local biodiversity.

Genomics have emerged as an effective tool for assessing diversity within and amongst populations. Swart et al. (2010) observed low differentiation among pig populations in Southern Africa using microsatellites. Heterozygosity levels ranged from 0.531 to 0.692 for commercial and indigenous breeds. The availability of the Porcine SNP60K BeadChip has opened new avenues of examining genetic diversity (Ramos et al., 2009) at a genome wide scale relative to that using microsatellite and other low-coverage markers. Mujibi et al. (2018) observed close clustering of Warthogs and Bush pigs using the Porcine SNP60K BeadChip. The Porcine SNP60K BeadChip has been used to infer on population structure and selection signatures in Chinese and European pig populations (Ai et al., 2013). Using this SNP panel in South African pig populations will provide comprehensive information on the genomic architecture of local, exotic and wild pig populations, which will guide future management and conservation. The objective of the present study was to provide a large-scale analysis of the genetic diversity and structure of South African local pig populations using the Porcine SNP 60K BeadChip. The study investigated diversity of South African pigs relative to global populations of 389 pigs consisting of villages and out-group pigs from South America, Europe, United States, and China amongst other countries.

## Materials and Methods

### Breeds/Populations Sampled

**FIGURE 1:**
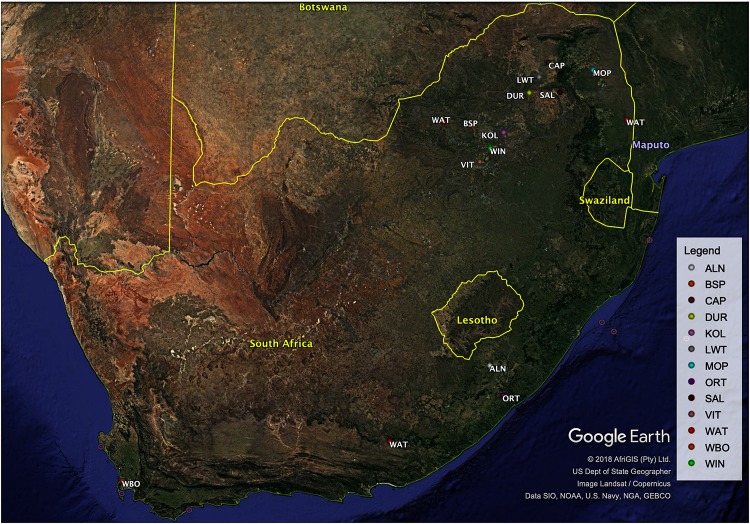
Map showing geographic locations of the 13 pig populations in the present study.

South African specimens were collected from a total of 234 samples from different production systems, representing village, intensively farmed populations in conservation units and free ranging populations. Village and non-descript pig populations were sampled from Alfred Nzo (ALN; *n* = 17) and Oliver Reginald Tambo (ORT; *n* = 22) districts in Eastern Cape province and Mopani (MOP; *n* = 27) and Capricorn (CAP; *n* = 25) districts in Limpopo province. Commercial pig breeds of Large White (LWT; *n* = 20), South African Landrace (SAL; *n* = 20) and Duroc (DUR; *n* = 20) were sampled from commercial farmers in Limpopo province. Indigenous populations Kolbroek (KOL; *n* = 20.) and Windsnyer (WIN; *n* = 20) were sampled from the Agricultural Research Council-Animal Production Institute in Pretoria, South Africa (Table 1). Vietnamese Potbelly breed (VIT; *n* = 5) was sampled from the Johannesburg Zoo and represents a breed that is endangered in Vietnam, its country of origin but has been raised in a conservation zoo in South Africa. European Wild Boar (*n* = 4), Warthogs (*n* = 31), and Bush pigs (*n* = 3) were sampled as representatives of the wild pig populations. The European Wild Boar and Bush pigs were sampled from the surrounding villages in the North-West whilst the Warthog samples were collected from geographically separated National Parks from North-West (*n* = 4), Eastern Cape (*n* = 3), and Limpopo (*n* = 24). The distribution of the sampled individuals is illustrated in Figure 1. Ear tissue samples were collected using the tissue sampling applicator gun while pliers were used to collect the hair samples according to standard procedures and ethical approval from ARC-Irene Animal Ethics committee (APIEC16/028).

**TABLE 1:** Population category and sample size of the 13 pig populations.

Category	Population	Code	*N*
Village	Mopani	MOP	27
Village	Capricorn	CAP	25
Village	Oliver Reginald Tambo	ORT	22
Village	Alfred Nzo	ALN	17
Commercial	Large White	LWT	20
Commercial	SA Landrace	SAL	20
Commercial	Duroc	DUR	20
Indigenous	Kolbroek	KOL	20
Indigenous	Windsnyer Type	WIN	20
Asian	Vietnamese Potbelly	VIT	5
Wild	Wild Boar	WBO	4
Wild	Warthog	WAT	31
Wild	Bush Pig	BSP	3

### Genotyping and Quality Control

DNA was extracted at the Agricultural Research Council-Biotechnology Platform from the ear tissue and hair samples using a commercially available Perkin Elmer Genomic DNA kit according to the manufacturer’s protocol. DNA concentration was quantified using the Qubit^®^ 2.0 Fluorometer. Gel electrophoresis (5%) was used to assess the quality and integrity of the DNA.

All 234 animals were genotyped using PorcineSNP60 v2 genotyping BeadChip (Illumina, United States) containing 62,163 SNPs with an average gap of 43.4 kb. Genotyping was done using the standard infinium assay at the ARC-Biotechnology Platform in South Africa. GenomeStudio version 2.0 (Illumina, United States) was used to process the genotype data, including raw data normalization, clustering and genotype calling. A final custom report was created to be able to generate a Plink *Ped* (Pedigree file) and *Map* (SNP panel file) for use in downstream analysis.

Golden Helix SNP Variation Suite (SVS) version 8.5 was used to update the SNPs marker file (Golden Helix Inc., 2016) based on the pig genome assembly (*Sus Scrofa* v10.2). Markers were then filtered to exclude SNPs located on the sex chromosomes. From this data set, Minor allele frequency (MAF) and deviation from Hardy–Weinberg equilibrium (HWE) were estimated per population for the 10 populations that excluded BSP, VIT, and WBO, which were left out due to small sample sizes. Additional quality control (QC) was also performed per population to remove SNPs with less than 85% call rate, MAF < 0.02 and HWE < 0.0001. The resultant filtered dataset was used to calculate observed (H_*O*_), and expected (*H*_*E*_) heterozygosities, inbreeding (*F*_*IS*_) and effective population size (*N*_*e*_).

Quality control was then performed overall population to remove SNPs with less than 85% call rate, MAF < 0.02 and HWE < 0.0001 and generate a dataset used for analysis of molecular variance (AMOVA) and *F*_*ST*_ analysis. Using this dataset, further QC filtered for SNPs in high LD (*r*^2^ = 0.2) and closely related individual [Identity By Descent (IBD) ≥ 0.45] to produce a filtered dataset used for population structure analysis using ADMIXTURE and Principle Component Analysis (PCA).

### Genetic Diversity Within Population

The MAF, *H*_*E*_ and *H*_*O*_ were calculated as measures of within population genetic variation using PLINK 1.07 (Purcell et al., 2007). In addition, inbreeding coefficient (*F*_*IS*_) was calculated on Golden Helix SNP Variation Suite (SVS) version 8.5 (Golden Helix Inc., 2016). Effective population size (*N*_*e*_) trends across generations were estimated based on a relationship between *r*^2^ (expected LD), *N*_*e*_ and *C* (recombination rate). SNeP software (Version 1.1) tool was used based on the following formula suggested by Corbin et al. (2012) using the equation:

$$N_{T\,\left(t\right)}=\frac{1}{\left(4\,f\,\left(C_{t}\right)\right)}\,\frac{1}{E\,\left[r_{a\,d\,j}^{2}|C_{t}\right]}-\alpha .$$

where:

*N*_*T(t)*_: Effective population size estimated *t* generations ago
*C*_*t*_: Recombination rate t generations ago
*r*_2adj_: Linkage disequilibrium estimation adjusted for sampling biasness
α: a constant.

The recombination rate was estimated by using the following formula proposed by Sved (1971):

$$f\,\left(c\right)=c\,\left[\frac{\left(1-\frac{c}{2}\right)}{{\left(1-2\right)}^{2}}\right].$$

The Bush pig, Vietnamese Potbelly and Wild Boar were excluded from the diversity within population analysis due to their small sample sizes. The few available samples were sampled from zoos and game reserves in the country where only few animals are often rescued and kept in conservation.

### Population Differentiation and Structure

Analysis of Molecular Variance (AMOVA) was used to determine the genetic variance within populations (*F*_*IS*_), among populations within group (*F*_*SC*_) and among groups (*F*_*CT*_) using ARLEQUIN v3.5 (Excoffier et al., 2005). The populations were categorized into villages, commercial, indigenous and wild populations and consisted of animals sampled in South Africa as well global populations from Burgos-Paz et al. (2013) which consisted of 389 genotypes of villages and out-group pigs from 24 countries of America (United States), South America (Mexico, Cuba, Guadeloupe, Guatemala, Costa Rica, Columbia, Ecuador, Peru, Brazil, Bolivia, Paraguay, Argentina, and Uruguay), Europe (Spain, Portugal, Italy, Poland, Hungary, Tunisia, Denmark, Holland, United Kingdom) and China. Variance components were also estimated for groups consisting of different categories, i.e., village and indigenous; indigenous and commercial; South African village and global villages; South African commercial and global commercial etc.

Principal Component Analysis (PCA) using SVS version 8.5 (Golden Helix Inc., 2016) and the eigenvector method was used to determine population clustering. ADMIXTURE version 1.20 (Alexander and Lange, 2011) was used to detect the most likely clusters (*K*) for the population. ADMIXTURE was run from *K* = 2 to *K* = 15. The number of potential genetic clusters (*K*) was tested from 1–15 to reassign each sample to its population of origin. The optimum *K*-value was that with the lowest cross-validation error value. Initially, all the 13 populations sampled from South Africa were included in the population structure analysis. After this the South African data set was merged to Porcine SNP60K genotype data from Burgos-Paz et al. (2013) described above.

Population pairwise *F*_*ST*_ values were estimated according to the formula of Weir and Cockerham (1984) implemented in the Golden Helix SNP Variation Suite (SVS) version 8.5 (Golden Helix Inc., 2016). Based on population pairwise *F*_*ST*_ values, PCA and ADMIXTURE based clustering, *F*_*ST*_ analysis per marker was estimated between pairs of highly differentiated populations of the village populations, indigenous populations and commercial breeds as well as amongst highly differentiated commercial breeds and wild populations. To reduce noise, an *F*_*ST*_ averaged smooth value was used to identify genomic regions differentiating pairs of populations. Manhattan plots of per marker *F*_*ST*_ values between pairs of populations were plotted against chromosomal coordinates using the porcine assembly (*Sus Scrofa* 10.2). Highly differentiating SNPs (*F*_*ST*_ ≥ 0.8) were subsampled and genes associated with these SNPs searched using genome browse including their associations with known QTLs in the pig genome based on the *Sus Scrofa* 10.2 on Ensembl^1^.

## Results

### Genotypes and Quality Control

The percentage of polymorphic and number of SNPs (*N*_*SNP*_) remaining after QC per population and overall is presented in Table 2. Two hundred and eleven individuals with a genotyping rate of 85% remained after QC. Windsnyer pigs had the highest percentage of informative markers (95%) after QC, whilst Warthog had the lowest at 82%. About 31,705 SNPs were removed leaving 30,458 polymorphic SNPs of the loci distributed over 18 autosomal chromosomes, which were used for AMOVA and *F*_*ST*_ analysis. After LD and IBD pruning, 23,345 SNPs and 176 individuals were used for the population structure analysis.

**TABLE 2:** Summary of the genetic diversity measures across South African Pig populations.

POP	*N*	%_*SNP*_	MAF ± SD	*N*_*SNP*_	*H*_*O*_ ± SD	*H*_*E*_ ± SD	*F*_*IS*_ ± SD	*P*-value
**MOP**	27	92	0.262 ± 0.149	52,925	0.299 ± 0.129	0.369 ± 0.131	0.198 ± 0.134	0.495
**CAP**	24	94	0.264 ± 0.147	54,078	0.332 ± 0.140	0.371 ± 0.126	0.117 ± 0.155	0.582
**ORT**	22	93	0.259 ± 0.153	52,238	0.315 ± 0.145	0.370 ± 0.130	0.163 ± 0.113	0.553
**ALN**	15	94	0.238 ± 0.157	53,580	0.336 ± 0.160	0.359 ± 0.134	0.056 ± 0.168	0.695
**LWT**	18	93	0.227 ± 0.161	49,773	0.358 ± 0.177	0.348 ± 0.144	0.023 ± 0.009	0.721
**SAL**	19	94	0.221 ± 0.162	49,191	0.372 ± 0.186	0.345 ± 0.144	0.052 ± 0.085	0.704
**DUR**	19	94	0.177 ± 0.168	40,632	0.359 ± 0.182	0.337 ± 0.147	0.067 ± 0.153	0.764
**KOL**	20	94	0.173 ± 0.167	39,560	0.364 ± 0.182	0.339 ± 0.144	0.051 ± 0.087	0.727
**WIN**	19	95	0.220 ± 0.164	47,402	0.385 ± 0.171	0.360 ± 0.134	0.056 ± 0.158	0.733
**WAT**	28	82	0.076 ± 0.109	3,967	0.188 ± 0.155	0.204 ± 0.151	0.398 ± 0.475	0.710

*%_*SNP*_ used to calculate MAF analysis; N_*SNP*_, the number of SNPs in the subset 62,163 SNP; *H*_*O*_, observed heterozygosity; *H*_*E*_, expected heterozygosity; SD, standard deviation; *F*_*IS*_, inbreeding co-efficient; MAF, minor allele frequency, *P* < 0.05.*

### Genetic Diversity Across Populations

Genetic diversity parameters among the 10 populations are summarized in Table 2. Warthog pigs had the lowest *H*_*O*_ (0.188 ± 0.155) and Windsnyer the highest (0.385 ± 0.171). Expected heterozygosity values ranged from 0.204 ± 0.151 from Warthog to 0.371 ± 0.126 for Capricorn. The highest inbreeding coefficient (*F*_*IS*_) was for Warthog at 0.398 ± 0.475 while the Duroc had the lowest and slightly negative value of −0.067 ± 0.153. *F*_*IS*_ values were positive for all village populations as well as Warthog suggesting some level of inbreeding within these populations. MAF was the highest in village population from Capricorn (0.264 ± 0.147) and the least in Warthog pigs (0.076 ± 0.109).

### Effective Population Size

**FIGURE 2:**
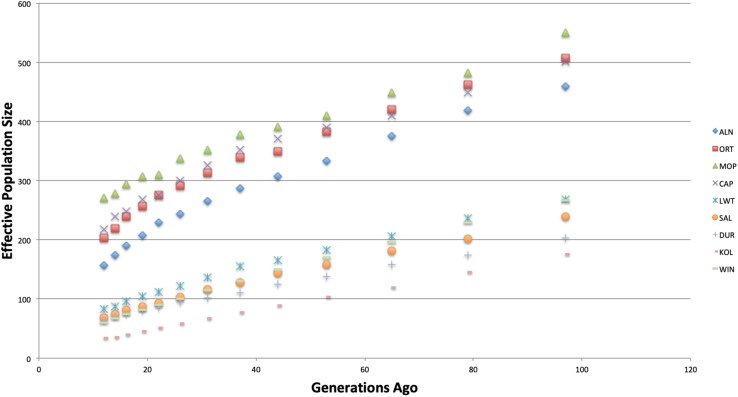
Average effective population size plotted against generation in the past.

Figure 2 shows trends in effective population size across all of the studied populations. The Warthog was excluded in this analysis because the number of polymorphic SNPs was not enough to generate results. Effective population size values are presented in Supplementary Table S1. There was a general decline in *N*_*e*_ across all the populations across generations. The indigenous and commercial populations had higher effective population size compared to the village populations. The Kolbroek had the lowest effective population size 12 generations prior.

### AMOVA

Genetic differentiation between populations is presented in Supplementary Table S2. The major proportion of the genetic variance was attributed to variation within South African populations with *F*_*IS*_ values ranging from 76.41 to 98.62%. Diversity within populations (*F*_*IS*_) in village populations from this study and those from Burgos-Paz et al. (2013) was 35.52% while variation among groups (*F*_*CT*_) was 62.35%. Diversity of South African commercial pigs was 76.41% within populations, 18.17% among populations within group and 5.42% among groups. When including the commercial breeds from Burgos-Paz et al. (2013), the diversity parameters changed to *F*_*IS*_ = 30.97%, *F*_*SC*_ = 8.31% and *F_*CT*_* = 60.72%. High *F*_*CT*_ (>60%) were observed in the category consisting of South African indigenous and Chinese indigenous (*F*_*CT*_ = 70.08%) as well as that consisting of the South African Wild Boar and the worldwide Wild Boar (*F*_*CT*_ = 73.58%).

### Population Structure

**FIGURE 3:**
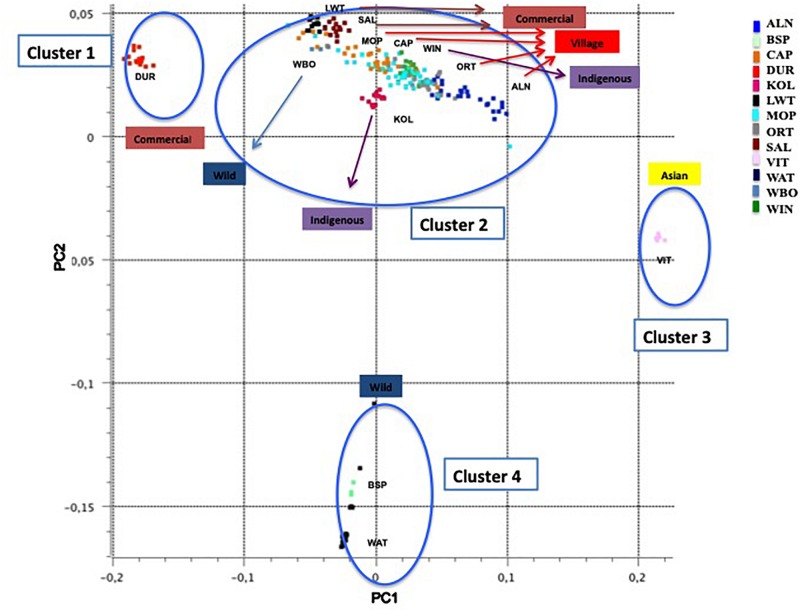
Principal Component Analysis based population clustering.

**FIGURE 4:**
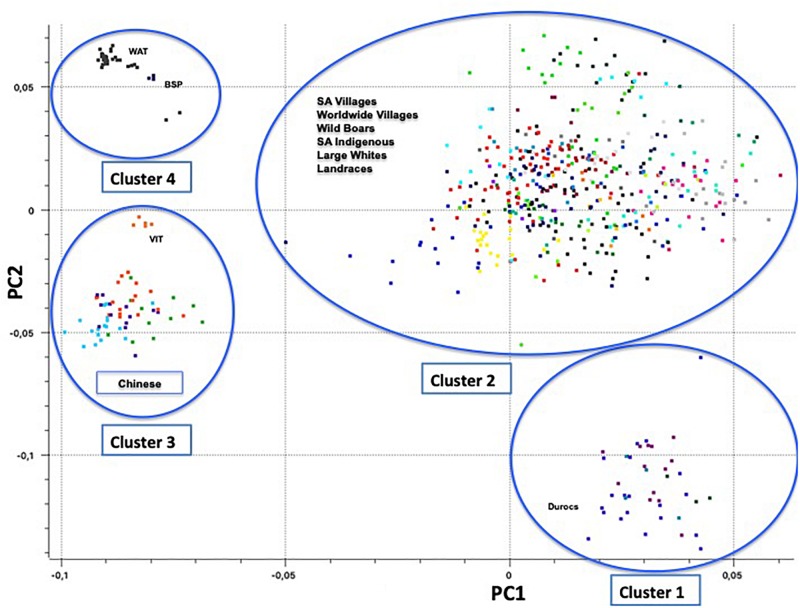
Principal Component Analysis based population clustering including Burgos-Paz et al. (2013) genotypes (22,430 SNPs).

Principal component one (PC1) and principal component two (PC2) explained approximately 30.7% and 11.8% of the total variation, respectively. The PCA of South African breeds yielded four main genetic clusters (Figure 3). The Duroc clearly separated from the Large White and South African Landrace that clustered together with the wild boar and village populations. The Warthog and the Bush pig clustered together as a third cluster whilst the fourth cluster consisted of Vietnamese potbelly sampled from the zoo. The PCA analysis using South African samples and those from Burgos-Paz et al. (2013) demonstrated the same clustering with all the village pigs grouping together with the Large White and Landraces separated from clusters of (i) Warthog and Bush pig, (ii) Chinese and Vietnamese breeds and (iii) Duroc (Figure 4).

**FIGURE 5:**
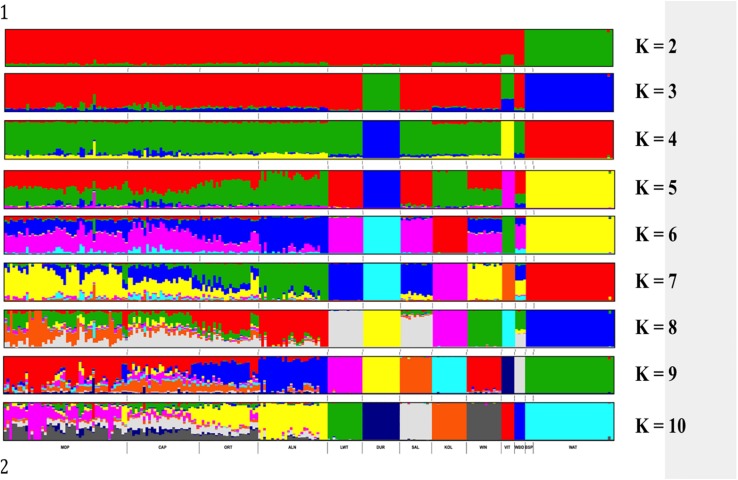
ADMIXTURE based clustering *K* = 2 – *K* = 10. Each individual is represented by a single column divided into *K* colored segments, where *K* is the number of clusters assumed with lengths proportional to each of the *K* inferred cluster.

Genetic structure of the South African breeds was further investigated using ADMIXTURE. The results presented in Figure 5 show the Warthog and Bush pig populations clustering together and clearly separated from the rest of the other populations at *K = 2*. Duroc separated from the rest of the populations at *K* = 3 followed by Vietnamese at *K* = 4. *K* = 4 clustered animals in the same way observed with PCA based clustering. Beyond *K* = 8, the genetic clusters of the commercial, indigenous, Asian and wild breeds are maintained whilst the added *K* is distributed within the village populations. *K* = 10 which was the optimal *K* (Supplementary Figure S1) with lowest CV (0.551) resulted in the eight distinct genetic clusters of commercial, indigenous, Asian and wild breeds plus highly admixed clusters consisting of all village pig populations from Limpopo and Eastern Cape provinces of South Africa.

### Population Differentiation

Population pairwise *F*_*ST*_ values are shown in Table 3. Low *F*_*ST*_ were observed between village populations with values ranging from 0.022–0.060 (*P <* 0.05) within South Africa and in global populations. The highest differentiation was found between Warthog and Duroc at *F*_*ST*_ = 0.481. Warthog and Kolbroek pigs showed the high differentiation at 0.468. All other populations had *F*_*ST*_ values above 0.282. The extent of differentiation between Warthog and all the other populations was high ranging from 0.312 (Warthog and Creole from Columbia) to 0.589 (Warthog and Vietnamese). Highest *F*_*ST*_ observed was between Vietnamese and Bush pig populations at 0.700 (Supplementary Table S3).

**TABLE 3:** Pairwise genetic differentiation (*F*_*ST*_ values) between 10 pig populations.

		MOP	CAP	ORT	ALN	LWT	DUR	SAL	KOL	WIN	WAT
**Villages**	**MOP**										
	**CAP**	0.022*									
	**ORT**	0.031*	0.026*								
	**ALN**	0.059	0.060	0.040*							
**Commercial**	**LWT**	0.091	0.073	0.096	0.130						
	**DUR**	0.134	0.126	0.143	0.174	0.183					
	**SAL**	0.094	0.073	0.099	0.132	0.120	0.194				
**Indigenous**	**KOL**	0.120	0.116	0.129	0.162	0.189	0.237	0.194			
	**WIN**	0.061	0.064	0.077	0.106	0.143	0.189	0.144	0.173		
**Wild**	**WAT**	0.282	0.306	0.314	0.350	0.433	0.481	0.435	0.468	0.410	

Significant levels: *P < 0.05.

### Per Marker Pairwise F_*ST*_, SNP Annotation and Association With Porcine QTLs

Per population, per marker pairwise *F*_*ST*_ values were computed for highly differentiated populations and are illustrated in Table 4, Supplementary Figure S2. SNPs. High *F*_*ST*_ values (≥0.8) where considered breed differentiating and the associated SNPs were functionally annotated for genes within a 1 MB region. Fixed SNPs (*F_*ST*_* = 1.0) where observed on chromosome 9 between Duroc and Warthog, on chromosome 12 between Koelbroek and Warthog and on chromosome 18 between Windsnyer and Warthog. For all the pairwise comparisons, 281 SNPs (*F*_*ST*_ ≥ 0.8) were detected (Supplementary Figure S2) with only 123 candidate genes within 1 MB of those SNPs. Pairwise comparison of village pigs from Alfred NZO, South Africa and Warthog yielded genes related to acute heat stress (*RPL18*) and inflammatory response (*IL17B* and *ARHGAP23*) as illustrated in Table 4 and Supplementary Figure S2a. Gene *ADGRB3* was in close proximity of SNPs *rs81353971, rs81353988, rs81353991, rs81297001*, and *rs81333295* that were of significant between Duroc and Warthog. Inflammatory response genes such as *ARHGAP23* were associated with the significant SNPs observed between Koelbroek, Large White and Windsnyer populations. For reproduction traits, genes *CD28*, *TCP11L2*, *TLK1*, *ATPB2, GPR137C, ZNF609*, *ARHGAP22, EPSTI1, GPR63, TCTE3, PTP4A2, ZSCAN20, CLU*, and *CACNA2D3* were observed within 14 significant SNPs on chromosomes 1, 2, 5, 6, 11, 14, and 15. Genes that had association with meat traits such as *DLX1*, *BRPF*1, *CLPTM1*, *FANCD2*, *SEC13*, *FHL3*, *FSTL5*, *CEP135, EXOC1, FOXO1, ASTN2, MYO18B, PLXNA1, DNAH2, HECTD2, TMEM39B, TXLNA, CSMD2, COL16A1, SCARA3, ZFAND3*, and *PTPRD* were also reported. Comparison with indigenous pigs showed genes that were associated with mastitis resistance (*ARHGAP39*, *ARPC4, PHC2*, and *BCL2L15*) and hair follicle development (*FOXN1*). A total of eight SNPs associated with growth traits (*ADGRB3*, *TSPAN*, and *ZFAND3*) were detected. *PTPN3* gene associated with immune response was observed between indigenous and Wild Boar. Wild Boar and Duroc comparison resulted in genes associated with adaptation (*HDAC1* and *GNAI3*).

**TABLE 4:** Most significant SNPs detected with *F*_*ST*_ analysis and the associated genes.

Population	SNP	Chr	Position	Genes	Function
**ALN and WAT**	rs81355030	1	84,376,735	*RPL18*	Acute heat stress (Newton et al., 2012)
	rs81367521	2	150,546,025	*IL17B*	Embryonic development, tissue regeneration and inflammation (Bie et al., 2017)
	rs81285672	12	23,638,629	*ARHGAP23*	Inflammatory response (Liu, 2015)

**DUR and**	rs81353971	1	49,024,494	*ADGRB3*	Growth traits (Emrani et al., 2017)
**WAT**	rs81353988	1	49,350,539	*ADGRB3*	
	rs81353991	1	49,392,902	*ADGRB3*	
	rs81297001	1	49,458,254	*ADGRB3*	
	rs81333295	1	49,592,586	*ADGRB3*	
	rs80946298	13	33,531,504	*DOCK3*	Induces axonal growth (Kimura et al., 2016)
	rs81444796	13	33,481,604	*DOCK3*	
	rs81478683	13	34,024,632	*IQCF3*	Conjunctival UV to auto fluorescence (Yazar et al., 2015)
	rs81478482	13	34,117,528	*ACY1*	Amino acid and heat shock protein (Martínez-Montemayor et al., 2008)
	rs81454214	15	107,134,695	*CD28*	Endometrial gene expression (Gu et al., 2014)

**KOL and WAT**	rs81341610	3	4,508,681	*LOC102160627*	Uncharacterized
	rs80993200	4	234,605	*ARHGAP39*	Milk production related and mastitis resistance (Wang et al., 2015)
	rs80851822	5	13,913,761	*POLR3B*	Residual feed intake (Gondret et al., 2017)
	rs80873063	5	13,940,475	*TCP11L2*	Regulated in small atretic follicles for healthy follicles (Hatzirodos et al., 2014a)
	rs80999600	5	66,998,856	*TSPAN9*	ADG (Fontanesi et al., 2014)
	rs80929588	5	67,092,749	*TSPAN9*	
	rs80883075	5	67,132,255	*TEAD4*	Regulation in organ size control and cell proliferation (Frankenberg et al., 2016)
	rs81385003	5	67,297,728	*ITFG2*	Disease resistance (Moioli et al., 2016)
	rs81285672	12	23,638,629	*ARHGAP23*	Inflammatory response (Liu, 2015)
	rs81325261	12	44,771,203	*FOXN1*	Regulation of hair follicle development (Song et al., 2017)
	rs80801871	13	33,170,033	*DOCK3*	Induces axonal growth (Kimura et al., 2016)
	rs80802886	13	33,202,454	*DOCK3*	
	rs81444784	13	33,306,071	*DOCK3*	
	rs81444796	13	33,481,604	*DOCK3*	
	rs80946298	13	33,531,504	*DOCK3*	
	rs81478683	13	34,024,632	*IQCF3*	Conjuctival UV to auto fluorescence (Yazar et al., 2015)
	rs335091311	15	148,461	*STAM2*	Residual feed intake (Gondret et al., 2017)
	rs80852223	15	77,232,829	*TLK1*	Decrease expression in the endometrium (Gray et al., 2006)
	rs80999734	15	77,318,065	*TLK1*	
	rs81453662	15	78,190,260	*DLX1*	Muscling and meat availability (Li et al., 2010)

**LWT and WAT**	rs81349766	1	182,224,202	*GPR137C*	Litter size (Sosa-Madrid et al., 2018)
	rs81296498	1	182,722,677	*DDHD1*	Lipid metabolism (Parker Gaddis et al., 2018)
	rs81349773	1	182,756,343	*DDHD1*	
	rs332395415	1	246,195,557	*ABCA1*	Mediates the transport of excess cholesterol (Schwartz et al., 2000)
	rs321979518	1	246,199,966	*ABCA1*	
	rs81383185	5	21,606,108	*RNF41*	Lipid rafts in immune signalling (McGraw and List, 2017)
	rs80820161	5	21,745,636	*STAT2*	Milk production (Salehi et al., 2015)
	rs80894897	5	21,727,701	*PAN2*	Fat yield (Suchocki et al., 2016)
	rs80940129	5	21,970,939	*BAZ2A*	Nutrition related (Cornelis and Hu, 2013)
	rs325229936	5	22,338,939	*MYO1A*	Coat color and pigmentation (Gutiérrez-Gil et al., 2007)
	rs81285672	12	23,6386,29	*ARHGAP23*	Inflammatory response (Liu, 2015)
	rs80854565	14	89,185,576	*ARHGAP22*	Fertility (Browett et al., 2018)
	rs80833618	14	89,227,581	*ARHGAP22*	
	rs80957034	14	89,255,703	*ARHGAP22*	
	rs80962102	14	89,309,115	*ARHGAP22*	

**SAL and WAT**	rs81395957	6	51,328,753	*NECTIN2*	Cell recognition and adhesion (Wang et al., 2010)
	rs81395929	6	51,427,663	*CLPTM1*	Marbling score (Lim et al., 2013)

**WIN and WAT**	rs81381252	4	65,339	*ZNF609*	Fertility (Hatzirodos et al., 2014b)
	rs81285672	12	23,638,629	*ARHGAP23*	Inflammatory response (Liu, 2015)
	rs81325261	12	44,771,203	*FOXN1*	Regulation of hair follicle development (Song et al., 2017)
	rs331955329	13	66,004,327	*MTMR14*	Reduced with age accelerates skeletal muscle aging (Romero-Suarez et al., 2010)
	rs80971430	13	66,026,240	*BRPF1*	Intramuscular fatty acid (Puig-Oliveras et al., 2016)
	rs80945527	13	66,104,857	*ARPC4*	Mastitis resistance (Grossi et al., 2014)
	rs80885182	13	66,270,725	*FANCD2*	Muscle weight (Lionikas et al., 2010)
	rs45430493	13	66,515,894	*SEC13*	Muscle weight (Lionikas et al., 2012)
	rs81248260	13	66,583,753	*ATPB2*	Heat stress on reproductive performance (Dash et al., 2016)
	rs81446451	13	66,668,301	*ATPB2*	
	rs81446497	13	66,691,206	*ATPB2*	
	rs81446475	13	66,725,741	*ATPB2*	
	rs81446484	13	66,777,686	*ATPB2*	
	rs81478601	13	66,795,578	*ATPB2*	

**IND and DUR**	rs80866460	4	106,698,421	*PTPN22*	Immune response (Lamsyah et al., 2009)
	rs81413279	9	79,010,742	*NXPH1*	DMI (Olivieri et al., 2016)
	rs81413279	9	79,010,742	*ABCB5*	Immune function (Lee et al., 2017)
	rs81306790	6	89,661,963	*PHC2*	Mastitis (Chen et al., 2015)
	rs80854994	4	106,719,032	*PTPN22*	Immune response (Lamsyah et al., 2009)
	rs80854994	4	106,719,032	*BCL2L15*	Mastitis (Chen et al., 2015)

**Villages and DUR**	rs81282695	6	94,442,844	*POU3F1*	Neurobehavioral functioning (Eusebi et al., 2018)
	rs81282695	6	94442844	*FHL3*	Carcass traits (Zuo et al., 2004, 2007)

**Villages and KOL**	rs81430450	11	24,063,007	*DNAJC15*	Feeding efficiency (Reyer et al., 2017a)
	rs81430450	11	24,063,007	*EPSTI1*	Fertility traits (Gaddis et al., 2016), fat deposition (Zhang et al., 2018)
**SAL&LWT and IND**	rs81232179	8	51,070,662	*FSTL5*	Meat quality (Ryu and Lee, 2016); skeletal muscle (Novianti et al., 2010)
	rs45431508	8	69,912,174	*CXCL8*	Pig disease (Wang et al., 2019)
	rs81400554	8	55,181,102	*CEP135*	Intramuscular fat (Hamill et al., 2012); milk production (Rui et al., 2013)
	rs81400554	8	55,181,102	*EXOC1*	Marbling score (Wu et al., 2016)
	rs81400740	8	63,119,376	*EPHA5*	Feed efficiency (Reyer et al., 2017b)
	rs81400500	8	52,213,568	*NPY5R*	Feed efficiency and fat deposition (Chen et al., 2018)
	rs81400500	8	52,213,568	*NPY1R*	Feed efficiency and fat deposition (Chen et al., 2018)
	rs81302014	8	69,950,857	*RASSF6*	Body conformation (Fang and Pausch, 2019)
	rs80904678	11	15,274,089	*FOXO1*	Meat quality and carcass traits (Ropka-Molik et al., 2018)
	rs81400500	8	52,213,568	*SLC7A11*	Feed efficiency (Vigors et al., 2016)
	rs81300083	9	78,940,661	*NXPH1*	DMI (Olivieri et al., 2016)

**IND and VIT**	rs81350922	1	257,096,974	*ASTN2*	Carcass weight in cattle (Júnior et al., 2016)
	rs80970078	14	43,524,181	*MYO18B*	Meat quality and carcass traits (Ropka-Molik et al., 2018)
	DRGA0006738	6	117,857,953	*NOL4*	Fatness (Li et al., 2011)
	rs80860919	1	64,018,444	*GPR63*	Fertility traits (Moran et al., 2017)
	rs80921694	13	73,023,057	*PLXNA1*	Meat quality (Martínez-Montes et al., 2016)
	rs81327396	12	53,063,765	*DNAH2*	Intramuscular fat (Luo et al., 2012); carcass weight (Kang et al., 2013)

**Villages and WBO**	rs81244815	2	50,167,007	*SWAP70*	Disease resistance (Ma et al., 2011; Zhang et al., 2018)
	rs81244815	2	50,167,007	*SBF2*	Fertility (Zhang et al., 2014); immune function (Ibeagha-Awemu et al., 2016)
	rs81401075	8	73,841,435	*FRAS1*	Sow reproductive traits (Fischer et al., 2015), feed efficiency (Messad et al., 2019)
	rs81401075	8	73,841,435	*NPY2R*	Obesity (Siddiq et al., 2007; Hunt et al., 2011)

**Villages and VIT**	INRA0003181	1	95,198,598	*SLC14A2*	Conformation traits (Le et al., 2017)
	rs81332040	6	45,777,816	*ZNF382*	Conformation traits (Le et al., 2017)
	INRA0045852	14	10,3086,988	*HECTD2*	Fat and meat quality traits (Piórkowska et al., 2018)
	rs80980839	4	93,722,493	*RHBG*	Ammonia transporter (Xiang et al., 2016)
	rs80971176	5	49,876,132	*SOX5*	Ear morphology (Edea et al., 2017)

**WBO and DUR**	rs80837120	1	565,627	*TCTE3*	Involved in spermatogenesis (Du et al., 2016)
	rs81389959	6	88,334,239	*PTP4A2*	Reproductive traits (Verardo et al., 2016); intramuscular fat (Martínez-Montes et al., 2016)
	rs81390106	6	88,751,010	*TMEM39B*	Intramuscular Fat (Cesar et al., 2018)
	rs81390106	6	88,751,010	*TXLNA*	Meat quality (Ropka-Molik et al., 2018)
	rs81390106	6	88,751,010	*HDAC1*	Altitude (Ban et al., 2015)
	rs81390106	6	88,751,010	*MARCKSL1*	Feed intake (Lindholm-Perry et al., 2016)
	rs81317489	6	89,640,457	*ZSCAN20*	Scrotal circumference (Sweett et al., 2018)
	rs81317489	6	89,640,457	*CSMD2*	Meat pH trait (Dong et al., 2014); Body weight (Yoshida et al., 2017)
	rs80894853	9	78,663,586	*NXPH1*	DMI (Olivieri et al., 2016)
	rs81389936	6	88,264,983	*COL16A1*	Carcass and meat quality traits (Choi et al., 2012)
	rs80790807	4	106,750,789	*PTPN22*	Immune response (Lamsyah et al., 2009)
	rs80790807	4	106,750,789	*BCL2L15*	Mastitis (Chen et al., 2015)
	rs80911350	14	11,345,116	*SCARA3*	Meat quality traits (Tizioto et al., 2015)
	rs80911350	14	11,345,116	*CLU*	Fertility (Kumar et al., 2015), intramuscular fat (de Jager et al., 2013)
	rs343528814	13	36,608,977	*CACNA2D3*	Reproductive traits (Smith et al., 2019); body width in gilts and sows (Rothschild, 2010), body weight traits (Borowska et al., 2017), altitude (Zhang et al., 2014)
	rs81478390	13	53,707,241	*RYBP*	Body conformation traits - body weight, body length, body height, and chest circumference (Zhou et al., 2016)
	rs81330369	9	7,449,894	*FCHSD2*	Milk production traits (Kemper et al., 2015)
	rs80975991	7	33,481,446	*ZFAND3*	Growth and carcass quality traits (Li and Kim, 2015)
	rs80855522	4	11,0552,282	*GNAI3*	Heat tolerance (Berihulay et al., 2019)
	rs80988392	1	213,780,848	*PTPRD*	Meat quality (Raschetti et al., 2013)
					

## Discussion

The Porcine SNP60K BeadChip was developed in 2009 (Ramos et al., 2009) and has been used to analyze genetic diversity and population structure in several pig populations (Ai et al., 2013; Burgos-Paz et al., 2013; Yang et al., 2017; Mujibi et al., 2018). This is the first report using the Porcine SNP60K BeadChip to explore diversity of domestic and wild pig populations covering the commercial, village, wild and conserved pigs farmed and reared in Africa. Pigs are possibly known to have reached Sub-Saharan Africa through the Nile corridor and later dispersed to the West-Central Africa (Blench, 2000). There are 541 pig breeds worldwide (Rischkowsky and Pilling, 2007) but the dominating commercial breeds in the pork industry are the Large White, Landrace, Duroc, Hampshire, Berkshire and Piétrain (Rothschild and Ruvinsky, 2010). The source of the improved breeds found in Southern Africa is believed to be the European settlers in 1600s (Krige, 1950; Blench and MacDonald, 2000; Swart et al., 2010). This was when Jan van Riebeeck brought some pigs to the Cape of Good Hope (Naude and Visser, 1994). The Large White, South African Landrace and the Duroc are the breeds mostly found and used in the commercial sector while the Kolbroek and Windsnyer are considered as indigenous and are mostly found in rural areas (Kem, 1993; Ramsay et al., 2000). The Vietnamese, Bush pig and Wild Boar populations constitute a small component of the genetic pool of pigs in the country often restricted to the game reserves and zoos.

The Porcine SNP60K BeadChip was designed using genomic resources from Western pig genomes (Ramos et al., 2009) and hence the number of SNPs after QC for the commercial population was higher (Table 2). The village populations had a higher number of polymorphic SNPs and moderate-high MAF compared to that of commercial pigs. Non-descript livestock populations including pigs are often observed to be highly diverse probably due to open mating systems and gene flow between populations. In South Africa similar observations of highly diverse and polymorphic populations were observed in village chicken populations (Khanyile et al., 2015), cattle (Makina et al., 2014), and village goats (Mdladla et al., 2016). The Warthog and other indigenous pigs were observed to be the least polymorphic and diverse which could be attributed to ascertainment bias as the Kolbroek, Windsnyer, Vietnamese Potbelly, Warthog and Bush pigs were not used in the development of the Porcine SNP60K BeadChip. Overall, the porcine SNP panel showed moderate MAF for the village, commercial and indigenous purebred pig populations such as the Windsnyer implying utility of the chip in the prevalent farmed pig populations of South Africa.

A study conducted by Swart et al. (2010) using microsatellite markers in various Southern African pig breeds revealed higher levels of diversity within population than was observed in this study for the same breeds (Table 2). High heterozygosity levels (0.61–0.75) were also reported by Halimani et al. (2012). In contrast to Swart et al. (2010) the Large White had the lowest diversity (*H_*o*_* = 0.358) compared to the South African Landrace (*H*_*o*_ = 0.372) and other breeds of the Duroc and Kolbroek. It must be noted that these previous studies used microsatellite markers that are highly polymorphic markers and cannot be compared to SNPs that are biallelic in nature. High gene diversity is therefore expected in microsatellites markers. However, results on genetic diversity from this study were comparable to other studies that used the Porcine SNP60K BeadChip in Chinese and Western pig populations (Ai et al., 2013).

The heterozygosity values for the indigenous pigs were relatively similar to those of the commercial pigs (Table 2). A lower diversity was expected for the commercial pigs as they are under selection while the indigenous pigs are known to be rich reservoirs of distinct alleles, coupled with presence of gene flow (Amills et al., 2012). However, the indigenous pig populations are also of very small flock sizes and often fragmented and restricted to specific farming communities and conservation units hence diversity was low. Small and fragmented populations and the possibility of natural selection due to disease and unfavorable climatic conditions could explain the genetic diversity observed in the village populations. The high inbreeding levels observed in the Warthog populations might have been promoted by its family structuring where pigs are organized into fragmented breeding and social units (Table 2). Somers et al. (1995) noted that a group of Warthogs consist of about 40% of adults with changes seasonally. The number of mature individuals is estimated to be between 2000 and 5000 in the Kruger National Park (Ferreira et al., 2013). The geographical separation of the three national parks from which the warthogs were sampled, could have created small and fragmented subpopulations leading to escalated *F*_*IS*_ values due to Wahlund effect. As expected, we found that the village pig populations of South Africa had high inbreeding values compared with other populations. The negative *F*_*IS*_ values for commercial and indigenous populations are reflective of their intensive production environment as individuals are outbred to avoid mating to close relatives.

The low levels of effective population size (*N*_*e*_) in the recent 12–22 generations for both commercial and indigenous populations are of concern (Supplementary Table S1). More so in the indigenous breeds since low levels of genetic diversity are likely to diminish overtime and increase the risk of extinction. The effective population of the Kolbroek of 34 at 12 generations ago is even lower than the minimum threshold *N*_*e*_ of 50 set by the FAO (2000). Franklin (1980) recommended a *N*_*e*_ of at least more than 500 while Willi et al. (2006) suggested *N*_*e*_ of more than 1,000 to maintain the evolutionary potential of any population. The genetic diversity of these populations will likely continue to be negatively impacted by the small number of founders and them being farmed in fragmented populations. Small effective population size of the Kolbroek might be due to pigs being raised in a research facility with limited boars and sows. Large White, Duroc and South African Landrace are commercial pigs that have undergone strong selection for meat and carcass traits thus resulting in small effective population sizes. Long-term sustainability of the populations might be compromised due to the small population size as it increases the effects of genetic drift and reduction in fitness traits (Frankham et al., 1998).

The high *F*_*IS*_ values observed within populations across breeds are similar to previous studies (SanCristobal et al., 2006; Swart et al., 2010; Gama et al., 2013; Edea et al., 2014). An overall AMOVA *F*_*IS*_ value of 93.95% was comparable to Halimani et al. (2012) value of 92.90% in indigenous pigs of Southern Africa. Diversity amongst South African populations that ranged from *F*_*CT*_ = 0.92 (village pigs) to *F*_*CT*_ = 5.42 (Commercial populations) might be due to gene flow between different populations within a sub-populations. Moderate diversity within population (i.e., *F*_*IS*_ ranging from 19.92 in the category consisting of South African Wild Boar and worldwide Wild Boar to *F*_*IS*_ = 35.52 in the categories consisting on South African villages and Worldwide villages) relative to elevated *F*_*CT*_ in the same categories implies a higher genetic variation distributed among groups from different geographic locations. This genetic variation observed amongst groups of the South African and Burgos-Paz et al. (2013) pig populations (i.e., *F*_*CT*_ = 62.35–73.58) is higher than the variation reported amongst Angora goats from South Africa, France and Argentina using 50K SNP BeadChip (Visser et al., 2016), which could be explained by limited exchange of breeding animals across geographic boundaries in the studied pig populations. The amongst population within groups diversity values ranging from *F*_*SC*_ = 0.46 for South African villages to *F*_*SC*_ = 18.17 for South African commercial demonstrates evidence of population sub-structure and genetic differentiation between the well-defined commercial and indigenous breeds relative to non-descript village populations that are characterized by weak population boundaries.

The PCA demonstrates the impact of domestication and geographic history on the clustering of populations. European populations as represented by Wild Boar, South African Landrace, and Large White, clustered together as expected (Figure 3). Considering the history that the Wild Boar is an ancestor to the domestic pigs of today, some gene flow may have remained from the Wild Boar in the domestic pigs (Giuffra et al., 2000). The clustering of the Wild Boars reflects a European ancestry of those populations within that cluster. The slight difference between the Wild Boar and domestic populations might have been due to geographic isolation and artificial selection. Geographic structures were evident amongst most of the pig populations that were aligned to production systems and their founder effects. The clustering of the Windsnyer and the village populations could be due to gene flow between indigenous breeds and village populations. Limpopo populations had a closer proximity to Large White and South African Landrace, and farmers in this region are more likely to buy pigs from commercial herds. The Large White and South African Landrace are also closer together as these are both European breeds. It was interesting that generally the village populations were closer to the Windsnyer and Kolbroek as these are both indigenous breeds in South Africa. Although not much is known about our indigenous breeds, different theories suggest that the Kolbroek might have far Eastern alleles while the Windsnyer is known to be dominant in other parts of Southern Africa like Mozambique, Zambia and Zimbabwe (Holness, 1973, 1991). The village populations and other Large Whites and Landraces from the global data set clustered together with the South African village, commercial and indigenous pigs demonstrating genetic similarities that could be aligned to founder effects and similarities in production systems.

The clustering of Duroc away from other commercial populations (Large White and South African Landrace) was expected. The Duroc breed was created in the United States with pigs of several ancestries, including African pigs (Porter, 1993). Studies conducted by Kotze and Visser (1996) and Swart et al. (2010) using the microsatellite markers on the Large White, South African Landrace and Duroc also reported similar results. The Large White and South African Landrace were more genetically similar when compared to the Duroc. The inclusion of global populations did not alter this clustering (Figure 4).

The distance of Vietnamese Potbelly population from the rest of the domestic pigs is clear evidence of independent domestication that took place between the European and Asian subspecies of the wild boar (Giuffra et al., 2000). The PCA including pigs genotyped from all over the world clearly shows the geographical effect of the populations as the Vietnamese Potbelly clustered in close proximity to the Chinese population.

ADMIXTURE *K* = 2 presented the first level of ancestry of the Suidae family representing *Phacochoerus africanus* (Warthog) and *Potamochoerus larvatus* (Bush pig) versus *Sus scrofa* (domesticated pigs including the Wild Boar) species (Figure 5). The presence of the Wild Boar genomic signature in the domestic pigs from *K* = 2 to *K* = 7 is not surprising (Figure 5). It is well documented that the domestic pigs diverged from each other and originated from the ancestral wild boars around 8,000–10,000 years ago (Giuffra et al., 2000; Laval et al., 2000; Larson et al., 2005). The Asian and European ancestral wild boars also originated from different subspecies thus the Vietnamese Potbelly diverged early (*K* = 2) from the rest of the domestic pig population. The results for the village populations showed high levels of admixture and weak between population sub-structuring. As opposed to pigs from the commercial sector that practices the intensive production systems, pigs in the villages are farmed under semi-intensive of free-range production systems, which might explain the admixture observed in this study. There is considerable indiscriminate crossbreeding that is taking place in village populations (Rege and Gibson, 2003). European and Asian pigs were used to improve the South African pig breeds but the actual contribution is unknown. Although phenotypically distinct from each other, the Bush pigs and warthogs clustered together which is suggestive of either common founder effect or selection pressures in the natural environments.

According to Wright (1978), *F*_*ST*_ estimation with values of less than 0.05 represents low differentiation while values between 0.05 and 0.15 represent a moderate genetic differentiation and those between 0.15 and 0.25 and beyond reflect highly differentiated populations. The low levels of genetic differentiation of the village populations from this study (Table 3) is consistent to pairwise *F*_*ST*_ values of Halimani et al. (2012) of village populations from Zimbabwe and South Africa. Most pig farmers from the villages practice free ranging or semi-controlled farming where there is continuous gene flow between populations within villages thereby explaining the low levels of population sub-structuring observed. Moderate *F*_*ST*_ values implies closer relationship between the South African Landrace and Large White and agrees with their breeding history, whereby the Landrace was developed from crossing the Large White from England and a Denmark indigenous. Greater genetic differentiation between the Warthog and the other pig populations (*F*_*ST*_ = 0.36–0.53) might be attributed to the (i) pressures of natural selection (ii) the separate histories of domestic and wild populations and (iii) the unique population dynamics of Warthogs that are known to live in clans of adult females, males and their offspring while maintaining minimal contacts with other clans (Cumming, 1975; Somers et al., 1994). In South Africa, Warthog populations are restricted to nature reserves thus creating a physical barrier and huge genetic differentiation between them and other pig populations. This will be in contrast to the greater interaction between village, commercial and indigenous populations. Low *F*_*ST*_ values between the villages in South African and village populations from South America (Supplementary Table S3) from Burgos-Paz et al. (2013) study, might be an indication that either common founder populations or similarities in production systems leading to common selection pressures. Ramírez et al. (2009) demonstrated that the African and South American pigs were derived from Europe and Far Eastern pigs. The very high genetic differentiation between the Vietnamese Potbelly and Bush pig agrees with the PCA and Admixture clustering.

Per marker pairwise *F*_*ST*_ were estimated between pairs highly differentiated populations which were from villages, commercial, indigenous, Asian and wild populations (Table 3). From the pairwise *F*_*ST*_, Warthog was found to be genetically different from the rest of the populations. The per marker pairwise *F*_*ST*_ analysis used a threshold of 0.8 and above to plot Manhattan graphs of the Warthog against the rest of the populations. From the SNPs showing a threshold of *F_*ST*_* ≥ 0.8, we looked at candidate genes and QTLs that can be associated with those SNPs to infer on traits that might have genetically differentiated the Warthog from Alfred Nzo, Duroc, Kolbroek, Large White, South African Landrace, and Windsnyer populations (Supplementary Figure S2).

Majority of the SNPs that were above the threshold between the Warthog and the rest of the populations were from chromosomes 1, 4, 5, 12, 13, and 15 (Table 4). Chromosomes 2 (Warthog vs. Alfred Nzo), 3 (Warthog vs. Kolbroek), 6 (Warthog vs. South African Landrace) and 14 (Warthog vs. Large White) seemed to be less common. Chromosome 1 with a total number of 12 SNPs was associated with reproduction and growth traits while the indigenous populations of Kolbroek and Windsnyer were differentiated on chromosome 4 that was also linked to reproduction and growth traits.

Warthog vs. Alfred Nzo had three SNPS (*F*_*ST*_ ≥ 0.8) that are associated with reproduction (*RPL18*, *IL17B*) and growth (*IL17B*, *ARHGAP23*) characteristics (Table 4). It is known that good nutrition is vital to be able to maximize growth performance. Genes *IL17B* and *ARHGAP23* are linked to inflammatory response (Liu, 2015; Bie et al., 2017) and the gastrointestinal tract where they play a role in the digestion and absorption of the nutrients. Inflammatory responses lead to reduction of feed intake, which in turn affects the growth of the animal (Liu, 2015). Selection on genes associated with inflammation in the populations of Warthog vs. Alfred Nzo might be an effect of the different diets these populations scavenge on. Medzhitov (2008) noted the inflammation response to be a protective mechanism from the stress and harmful environment.

Growth linked genes *ADGRB3*, and *ACY1* were dominant in differentiating Warthog vs. Duroc populations with an overall total of 10 SNPs. Emrani et al. (2017) associated *ADGRB3* to body weight traits in the broiler chickens. The association of *ADGRB3* gene to Duroc rather than Large White or South African Landrace breeds might be linked to the higher percentage of intramuscular fat in Duroc compared to the other two commercial breeds (De Vries et al., 2000). Mature males of Warthog can also reach up to 100 kg and possesses good meat and carcass qualities (Hoffman and Sales, 2007).

A total number of 20 significant SNPs (*F*_*ST*_ ≥ 0.8) were linked to the Warthog vs. Kolbroek populations. Growth traits were associated with five of the SNPs between Warthog vs. Kolbroek. Indigenous Kolbroek are reported to be smaller in size when compared to commercial breeds such as Large White (Chimonyo et al., 2005). Kutwana et al. (2015) reported no significant difference (*P > 0.05*) between the Kolbroek and Large White populations that had higher fat percentages when compared to the other commercial breeds (Nicholas, 1999).

Chromosome 13 was also highly notable with significant SNPs differentiating Warthog vs. Kolbroek and Warthog vs. Windsnyer. Only two SNPs appeared for Warthog vs. South African Landrace and were on chromosome 6. The Warthog vs. Windsnyer had a total of fourteen SNPs differentiating them. The identification of BRPF1 gene in the Warthog vs. Windsnyer populations is an important observation as this gene is associated with the intramuscular fat (IMF). When it comes to the value and taste of the pork meat, intramuscular fat is an important characteristic because meat that is high in IMF tends to be juicy and tender (Eikelenboom et al., 1996; de Koning et al., 1999). The gene *ATPB2* associated with six significant SNPs is linked to heat stress and reproductive performance (Dash et al., 2016). Heat stress might result in poor reproduction for both sows and boars. Pigs cannot sweat and this makes them sensitive to high environmental temperatures making and of concern particularly to commercial pig farmers (Ross et al., 2015).

Genes linked to immune response and mastitis were observed in Indigenous vs. Duroc comparisons. *PTPN22* gene on chromosome 4 has a regulatory effect on T- and B- cell activation in immune response (Lamsyah et al., 2009). *PTPN22* plays a role in susceptibility to tuberculosis. Pigs are generally natural hosts of mycobacterial infections (de Lisle, 1994). Porcine TB has been reported in South Africa where infections are commonly via infected cattle fecal matter fed to piglets as well as interactions with wild pigs (Muwonge et al., 2012). *NXPH1* gene is associated with DMI (dry matter intake) in cattle (Olivieri et al., 2016). Both *PTPN* and *NXPH1* genes were fixed in the Duroc implying natural selection of the Duroc when compared to both indigenous and Wild Boars. Breeds in the commercial sector are mainly selected for growth, carcass and meat quality traits. The indigenous and village population on the other hand has not been systematically selected for such traits.

The *NPY5R* located on chromosome 8, was associated with feed efficiency and fat deposition. This gene was also reported in Jinhua and Rongchang pigs that belong to Chinese breeds (Chen et al., 2018). Fat deposition genes observed in Indigenous vs. Vietnamese, Villages vs. Kolbroek and South African Landrace with Large White vs. Indigenous are evidence in agreement with suggestions that Kolbroek and other indigenous pigs tend to carry their weight in their bellies and backs (Hoffman et al., 2005). Hoffman et al. (2005) also reported breed type and diet to have an influence on the composition of the meat. This study therefore presented a diverse genomic architecture of South African pigs with differentiating selection pressures for meat and carcass quality traits in the different pigs raised in diverse production systems.

## Conclusion

Overall, the study demonstrated the utility of the Porcine SNP60K BeadChip in elucidating genetic diversity and population genomic structure of South African pig populations relative to other global populations. Village pigs demonstrated distinctiveness from other domestic and commercial populations within South Africa and when compared to global populations. The study provided baseline knowledge with regards to the genetic diversity of the domestic and wild pig populations of South Africa, which is a prerequisite for population/breed characterization, utilization and conservation. A more in-depth analysis of patterns of genetic variations is required to get more insight into factors shaping genetic diversity of these populations.

## Data Availability Statement

The datasets generated for this study can be found in Dryadhttps://doi.org/10.5061/dryad.b0t10b0.

## Ethics Statement

Ear tissue samples were collected from pigs using the Tissue Sampling Applicator Gun while pliers were used to collect the hair samples according to standard procedures and ethical approval from ARC-Irene Animal Ethics committee (APIEC16/028).

## Author Contributions

NH collected samples, analyzed the data, and wrote the draft manuscript. FM, PS, and ED designed the experiment and sourced funding. KH analyzed the genomic data for the experiment. FM, PS, and ED coordinated the conduct of the study and writing of manuscript and revisions. All authors read and approved the manuscript.

## Conflict of Interest

The authors declare that the research was conducted in the absence of any commercial or financial relationships that could be construed as a potential conflict of interest.

## Footnotes

1 www.ensembl.org

## Supplementary Material

FIGURE S1 Cross validation plot for inferring the number of *K* populations in the analysis of population structure.

FIGURE S2 Genome-wide Manhattan plot of *F*_*ST*_ among the pig populations of **(a)** Alfred Nzo and Warthog, **(b)** Duroc and Warthog, **(c)** Kolbroek and Warthog, **(d)** Large White and Warthog, **(e)** South African landrace and Warthog, **(f)** Windsnyer and Warthog, **(g)** Indigenous and Duroc, **(h)** Villages and Duroc, **(i)** Villages and Kolbroek, **(j)** South African Landrace with Large White and Indigenous, **(k)** Indigenous and Vietnamese, **(l)** Villages and Wild Boar, **(m)** Villages and Vietnamese, **(n)** Wild Boar and Duroc. The solid lines indicate the *F*_*ST*_ ≥ 0.8 thresholds.

TABLE S1 Average effective population size estimates across generations for the different populations analyzed.

TABLE S2 Partitioning of genetic variance for the different populations analyzed.

TABLE S3 *F*_*ST*_ between SA populations and genotypes from Burgos-Paz et al. (2013).
