# Supplementary figures and images for: Genome Wide Assessment of Genetic Variation and Population Distinctiveness of the Pig Family in South Africa
Source: Front Genet. 2020 May 7;11:344. doi: 10.3389/fgene.2020.00344 (PMC7221027; doi:10.3389/fgene.2020.00344)

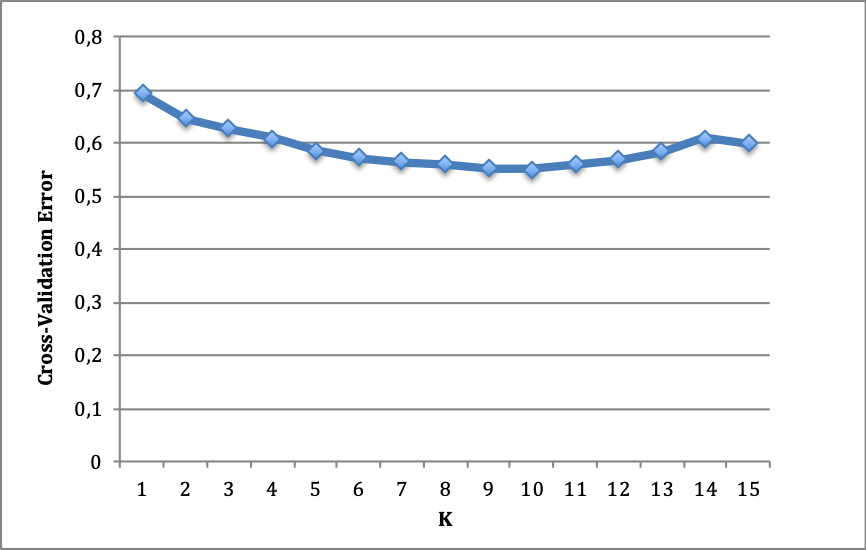


**Figure S1 | Cross validation plot**

Supplement: FIGURE S1 — Cross validation plot for inferring the number of K populations in the analysis of population structure. [file Data_Sheet_1.DOCX]

a)

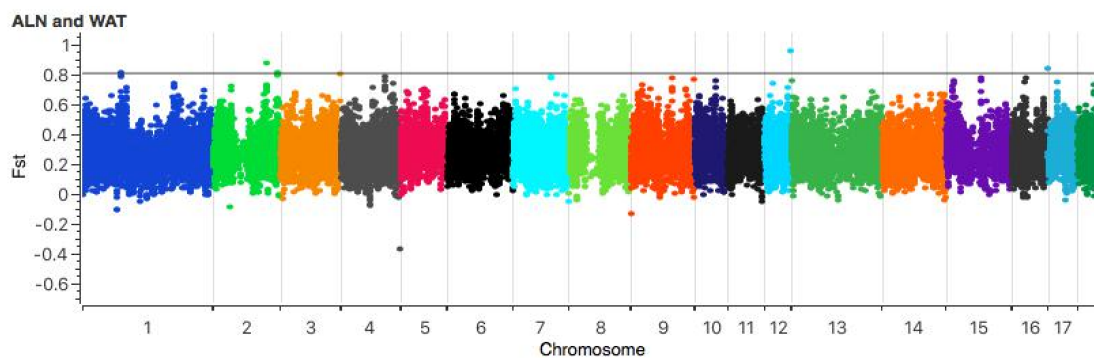

b)

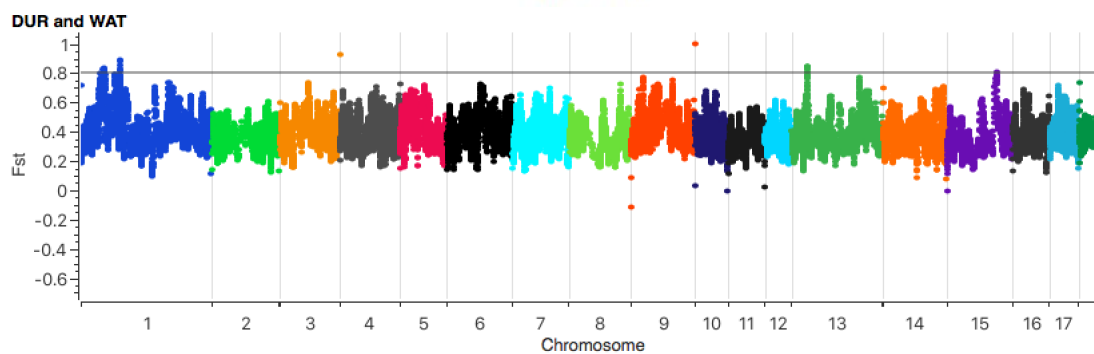

c)

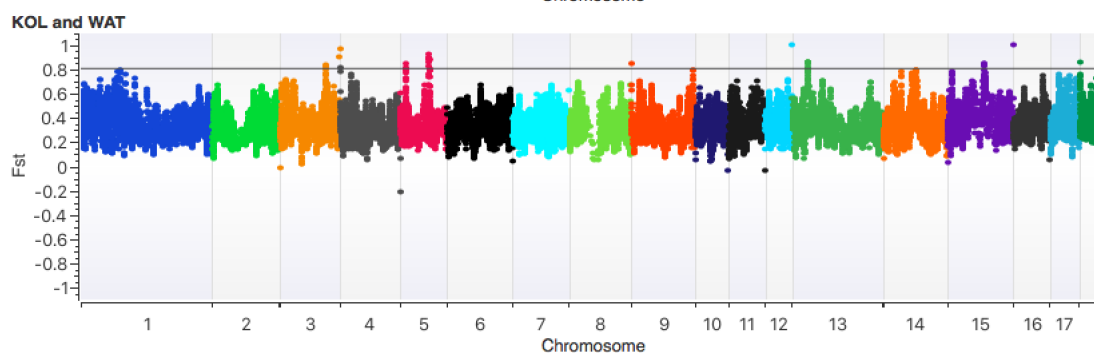

d)

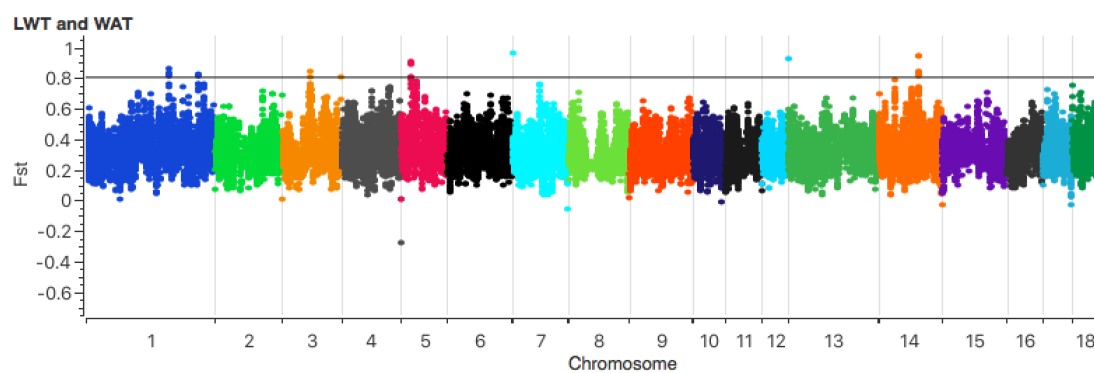

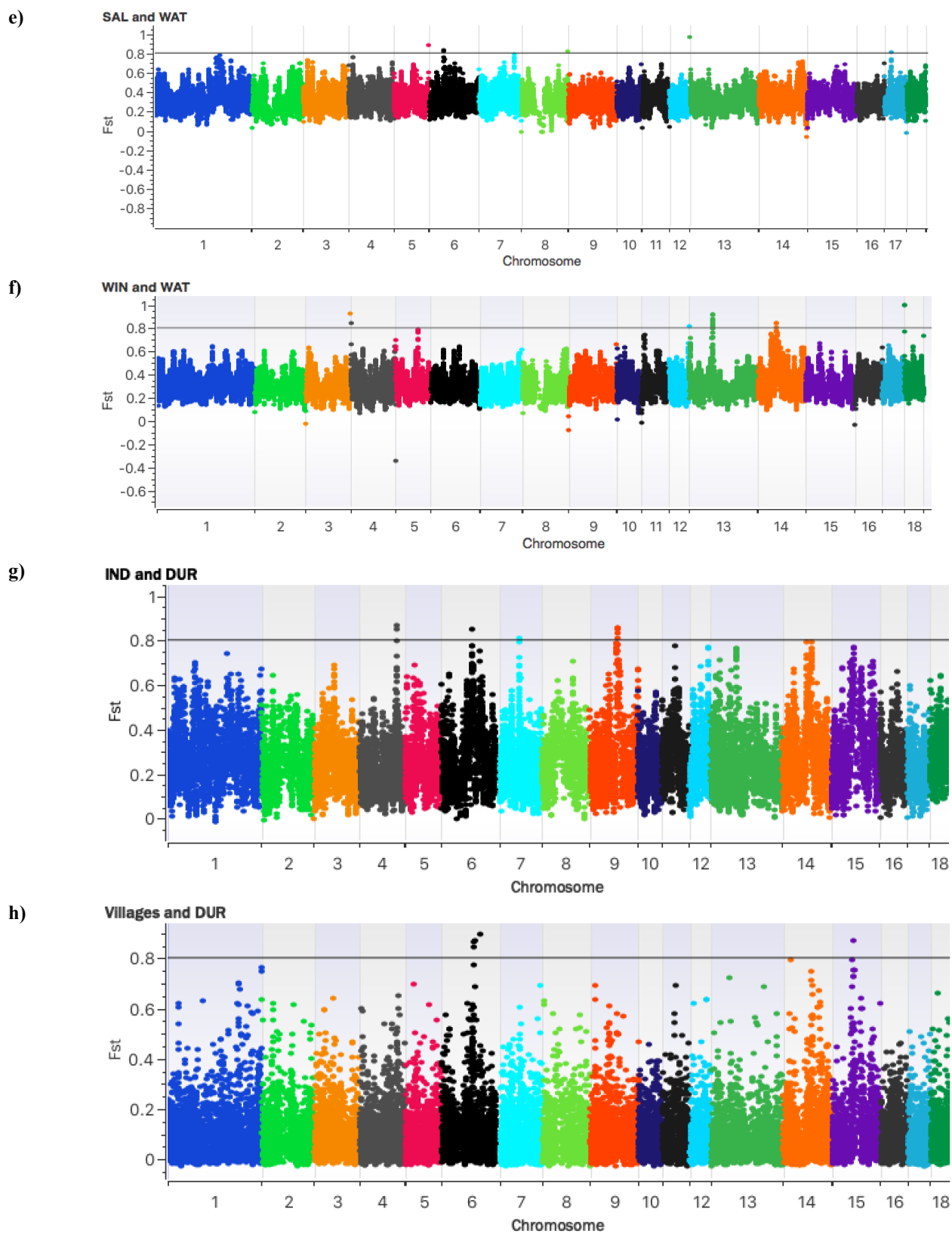

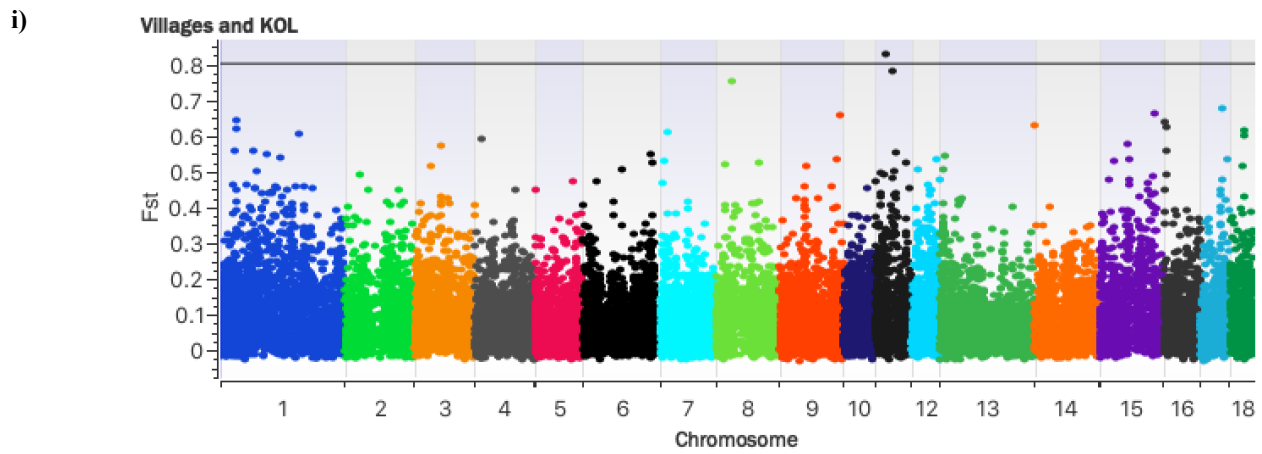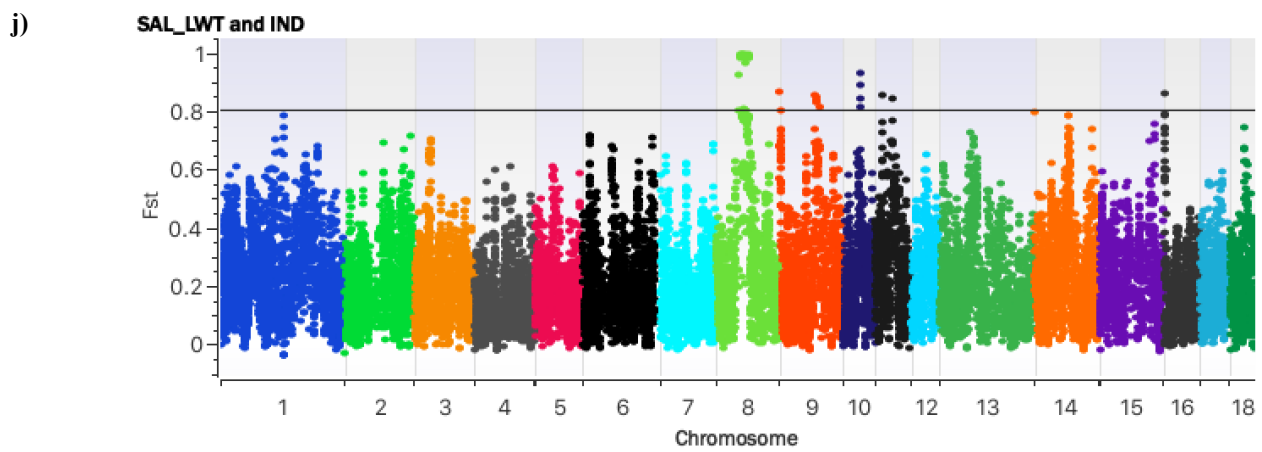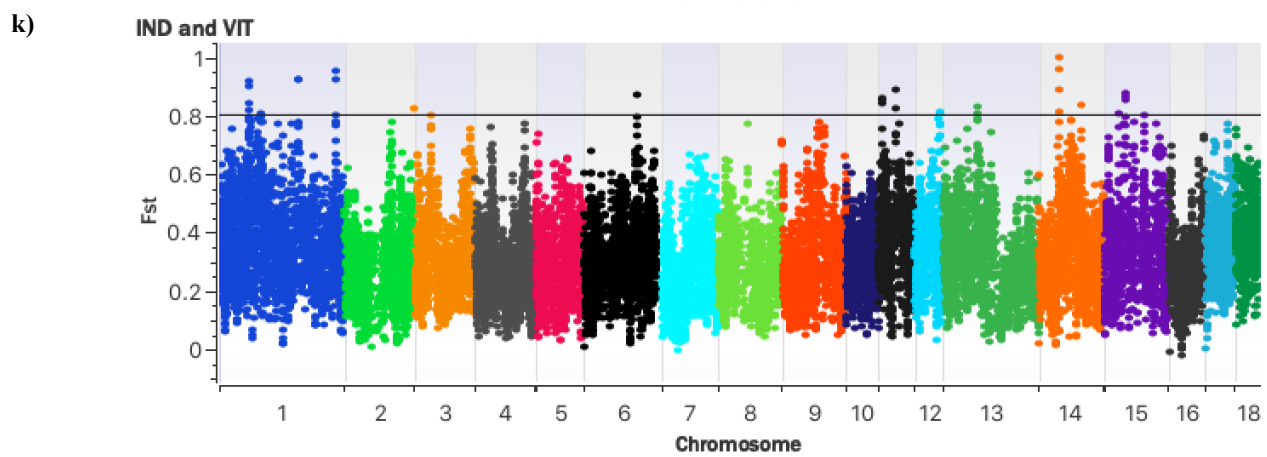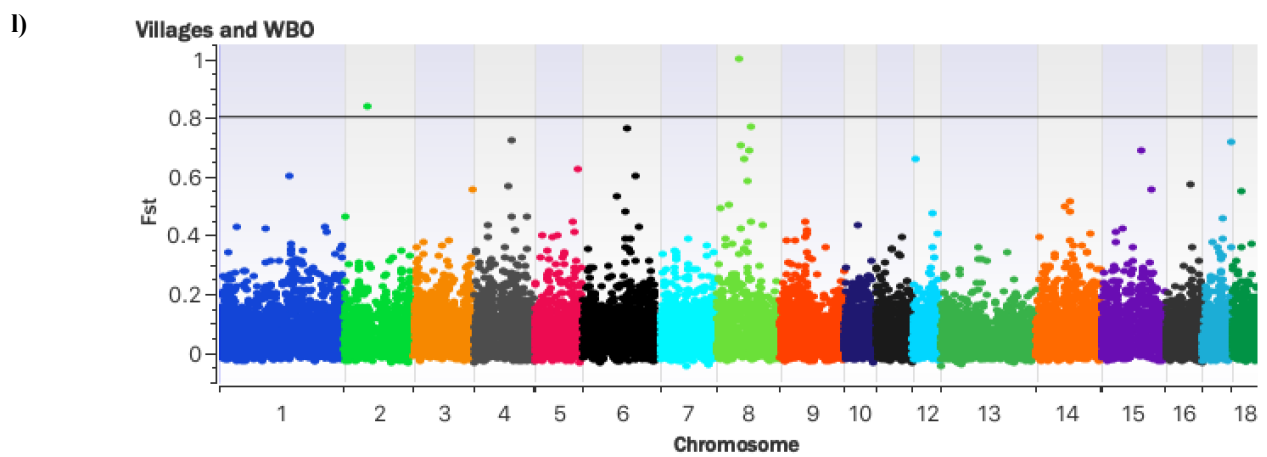

m)

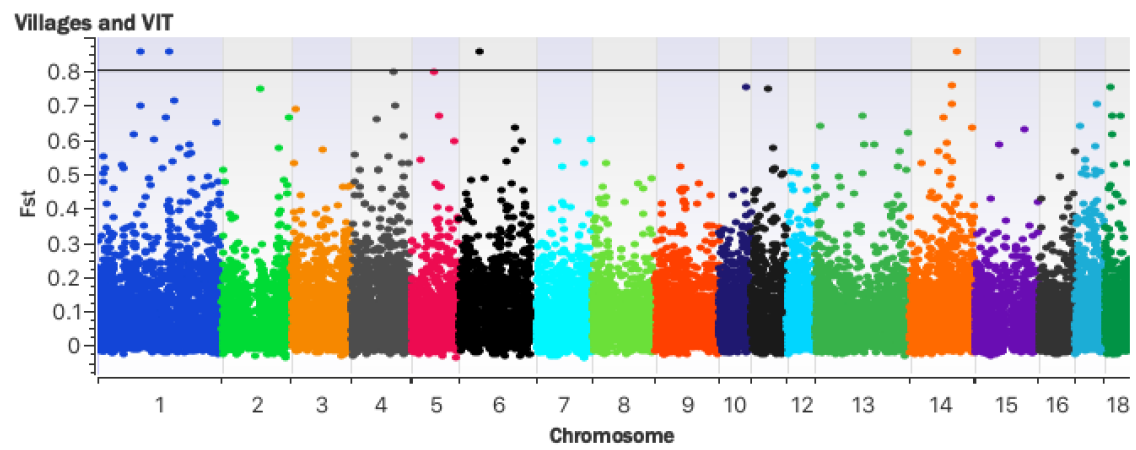

n)

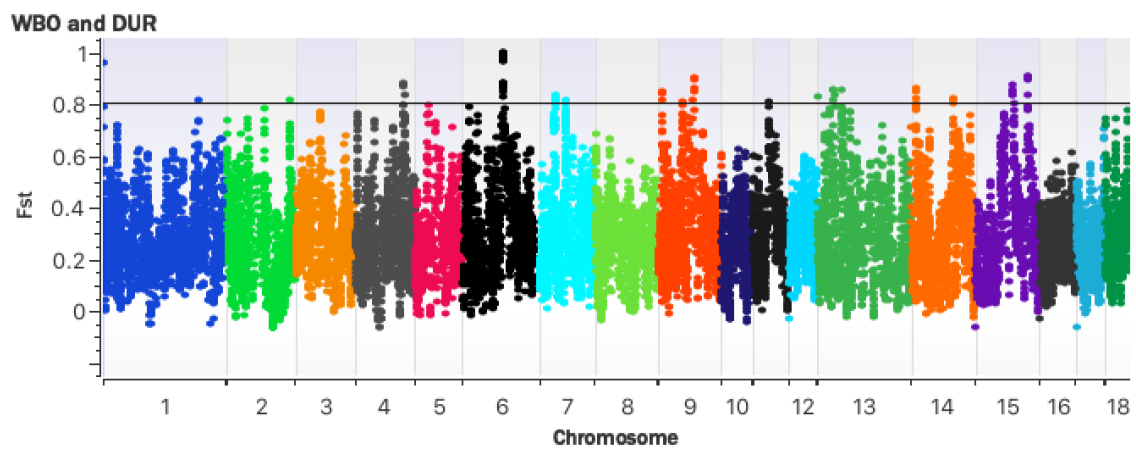

Supplement: FIGURE S2 — Genome-wide Manhattan plot of FST among the pig populations of (a) Alfred Nzo and Warthog, (b) Duroc and Warthog, (c) Kolbroek and Warthog, (d) Large White and Warthog, (e) South African landrace and Warthog, (f) Windsnyer and Warthog, (g) Indigenous and Duroc, (h) Villages and Duroc, (i) Villages and Kolbroek, (j) South African Landrace with Large White and Indigenous, (k) Indigenous and Vietnamese, (l) Villages and Wild Boar, (m) Villages and Vietnamese, (n) Wild Boar and Duroc. The solid lines indicate the FST ≥ 0.8 thresholds. [file Data_Sheet_2.PDF]
