# Supplementary material for: Genome Wide Assessment of Genetic Variation and Population Distinctiveness of the Pig Family in South Africa
Source: Front Genet. 2020 May 7;11:344. doi: 10.3389/fgene.2020.00344 (PMC7221027; doi:10.3389/fgene.2020.00344)
Supplement: TABLE S1 — Average effective population size estimates across generations for the different populations analyzed. [file Table_1.DOCX]

| **Table S1 \| Average effective population values** | | | | | | | | | |
| --- | --- | --- | --- | --- | --- | --- | --- | --- | --- |
| **Generations** | **ALN** | **ORT** | **MOP** | **CAP** | **LWT** | **SAL** | **DUR** | **KOL** | **WIN** |
| 12 | 157 | 204 | 271 | 218 | 83 | 69 | 65 | 34 | 60 |
| 14 | 174 | 220 | 278 | 239 | 88 | 75 | 71 | 36 | 67 |
| 16 | 191 | 239 | 294 | 248 | 96 | 82 | 75 | 40 | 74 |
| 19 | 208 | 257 | 307 | 269 | 105 | 88 | 81 | 46 | 82 |
| 22 | 229 | 276 | 310 | 276 | 112 | 94 | 86 | 51 | 90 |
| 26 | 244 | 292 | 338 | 301 | 123 | 104 | 95 | 59 | 101 |
| 31 | 265 | 313 | 352 | 326 | 137 | 116 | 102 | 68 | 115 |
| 37 | 288 | 339 | 379 | 352 | 156 | 128 | 111 | 78 | 131 |
| 44 | 308 | 350 | 391 | 371 | 166 | 144 | 125 | 89 | 150 |
| 53 | 333 | 383 | 410 | 390 | 183 | 159 | 138 | 103 | 170 |
| 65 | 376 | 420 | 450 | 411 | 207 | 181 | 158 | 120 | 196 |
| 79 | 419 | 463 | 483 | 449 | 237 | 202 | 175 | 145 | 230 |
| 97 | 460 | 508 | 551 | 501 | 268 | 240 | 204 | 176 | 267 |
| 120 | 519 | 579 | 619 | 569 | 318 | 279 | 236 | 211 | 330 |
| 149 | 606 | 659 | 701 | 650 | 375 | 334 | 284 | 256 | 381 |
| 186 | 665 | 728 | 763 | 721 | 438 | 400 | 342 | 319 | 461 |
| 233 | 769 | 845 | 878 | 834 | 525 | 478 | 411 | 391 | 544 |
| 292 | 900 | 1006 | 1009 | 977 | 622 | 573 | 504 | 478 | 698 |
| 365 | 1066 | 1147 | 1185 | 1103 | 781 | 701 | 595 | 577 | 827 |
| 453 | 1288 | 1288 | 1327 | 1264 | 880 | 809 | 724 | 685 | 956 |
| 552 | 1483 | 1553 | 1573 | 1469 | 1037 | 962 | 816 | 860 | 1132 |
| 657 | 1691 | 1693 | 1685 | 1646 | 1222 | 1071 | 993 | 1010 | 1345 |
| 759 | 1981 | 1946 | 1897 | 1810 | 1422 | 1241 | 1080 | 1063 | 1549 |
| 845 | 2005 | 2220 | 2213 | 1997 | 1547 | 1423 | 1260 | 1386 | 1628 |
| 914 | 2365 | 2148 | 2188 | 2220 | 1603 | 1302 | 1412 | 1248 | 1685 |
