# Supplementary material for: Genome Wide Assessment of Genetic Variation and Population Distinctiveness of the Pig Family in South Africa
Source: Front Genet. 2020 May 7;11:344. doi: 10.3389/fgene.2020.00344 (PMC7221027; doi:10.3389/fgene.2020.00344)
Supplement: TABLE S2 — Partitioning of genetic variance for the different populations analyzed. [file Table_2.DOCX]

| **Table S2 \| Partitioning of genetic variability** | | | |
| --- | --- | --- | --- |
| **Population** | **Variance Component (%)** | | |
|  | **Among groups (F_CT_)** | **Among population within groups (F_SC_)** | **Within population (F_IS_)** |
| **All Population** | - | 467.04 (6.04) | 7 254.77 (93.95) |
| **Villages** | 70.68 (0.92) | 35.41 (0.46) | 7 603.74 (98.62)^*^ |
| **Commercial** | 231.11 (5.42) | 774.63 (18.17) | 3 257.69 (76.41) |
| **Indigenous** | 181.15 (2.39) | 308.33 (4.07) | 7 088.76 (93.54) |
| **Villages and Indigenous** | 95.67 (1.26) | 186.24 (2.45) | 7 308.75 (96.29)^*^ |
| **Indigenous and Commercial** | 81.06 (1.05) | 569.79 (7.37) | 7 078.81 (91.58) |
| **Villages and ^#^Worldwide Villages** | 6 733.57 (62.35)^**^ | 229.50 (2.13) | 3 836 .44 (35.52) |
| **Commercial and ^##^Worldwide Commercial** | 6 512.97 (60.72)^**^ | 891.55 (8.31) | 3 322.49 (30.97) |
| **Indigenous and ^###^Chinese Indigenous** | 7 379.29 (70.08)^**^ | 791.54 (7.52) | 2 358.38 (22.40) |
| **Wild Boar and ^####^Worldwide Boar** | 7 947.70 (73.58)^**^ | 701.77 (6.50) | 2 152.06 (19.92) |

**Significant: ^*^*P* < 0.050; ^**^*P* < 0.001**

**^#^ = Argentina Villages; ^##^ = Duroc, Landrace, Large White from Denmark, Holland and USA; ^###^ = Jiangquhai, Jinhua, Xiang and Meishan; ^####^ = Western Wild Boars**
